# Identifying freshwater mussels (Unionoida) and parasitic glochidia larvae from host fish gills: a molecular key to the North and Central European species

**DOI:** 10.1002/ece3.220

**Published:** 2012-04

**Authors:** Alexandra Zieritz, Bernhard Gum, Ralph Kuehn, Juergen Geist

**Affiliations:** 1 Aquatic Systems Biology Unit, Department of Ecology and Ecosystem Management, Technische Universität MünchenMühlenweg 22, 85354 Freising, Germany; 2 Molecular Zoology Unit, Chair of Zoology, Department of Animal Science, Technische Universität MünchenHans-Carl-von-Carlowitz-Platz 2, 85354 Freising, Germany

**Keywords:** Host–parasite interactions, morphological variability, PCR-RFLP, species identification, Unionidae, wildlife management

## Abstract

Freshwater mussels (order Unionoida) represent one of the most severely endangered groups of animals due to habitat destruction, introduction of nonnative species, and loss of host fishes, which their larvae (glochidia) are obligate parasites on. Conservation efforts such as habitat restoration or restocking of host populations are currently hampered by difficulties in unionoid species identification by morphological means. Here we present the first complete molecular identification key for all seven indigenous North and Central European unionoid species and the nonnative *Sinanodonta woodiana*, facilitating quick, low-cost, and reliable identification of adult and larval specimens. Application of this restriction fragment length polymorphisms (RFLP) key resulted in 100% accurate assignment of 90 adult specimens from across the region by digestion of partial ITS-1 (where ITS is internal transcribed spacer) polymerase chain reaction (PCR) products in two to four single digestions with five restriction endonucleases. In addition, we provide protocols for quick and reliable extraction and amplification of larval mussel DNA from complete host fish gill arches. Our results indicate that this new method can be applied on infection rates as low as three glochidia per gill arch and enables, for the first time, comprehensive, large-scale assessments of the relative importance of different host species for given unionoid populations.

## Introduction

Freshwater mussels (order Unionoida) are among the most critically endangered groups of animals worldwide (Lydeard et al. 2004; Geist 2011). In North America alone, about 36 species (10%) are already presumed extinct (Neves et al. 1997), and three of the eight unionoid species of the North and Central European region are currently listed in the IUCN Red List of Threatened Species (IUCN 2011). As freshwater mussels have important ecosystem functions such as particle filtration and processing, nutrient release, and sediment mixing (Vaughn and Hakenkamp 2001), their decline can profoundly affect ecosystem processes in aquatic habitats (Geist and Auerswald 2007).

Accurate identification of mussel species and populations is a prerequisite for developing and carrying out measures for these animals’ conservation. However, due to a high degree of morphological variability within (Zieritz and Aldridge 2009; Zieritz et al. 2010; Zieritz and Aldridge 2011) and convergences between unionoid species (Watters 1994), taxonomic identification by means of morphological and, in particular, shell characters, is often unreliable. Molecular techniques such as DNA barcoding or identification keys based on restriction fragment length polymorphisms (RFLP) can provide a powerful alternative tool for identification of adult mussels (e.g., Kneeland and Rhymer 2007; Boyer et al. 2011). At the same time, such a molecular approach can prove helpful for a reliable and quick identification of these animals’ larvae (glochidia). During this early life stage, unionoids live as obligate parasites upon freshwater fish gills or fins; a unique life-history trait that is believed to have evolved as a means of dispersal of these otherwise relatively immobile animals (Kat 1984; Wächtler et al. 2001). Due to their small size (<1 mm in diameter), morphological identification of glochidia requires examination by scanning electron microscopy (e.g., Pekkarinen and Englund 1995). Inconsistencies in characters such as shape alteration of encysted glochidia can lead to misidentifications by morphology (Wiles 1975; O’Brien et al. 2003).

A reliable method for identifying glochidia is particularly important as decline or loss of host fish populations can profoundly affect mussel populations (Bogan 1993; Lyons et al. 2007). Only with a good understanding of the fish species used by respective mussel species and populations, can we develop appropriate conservation measures and perform captive breeding programs for these important organisms. Unfortunately, current knowledge on suitable host fish species and, in particular, relative importance of the respective host species used by different mussel populations is insufficient. While it has been established that some unionoids can use a range of host species (e.g., *Anodonta anatina* [Linnaeus 1758]; Wächtler et al. 2001) and others are quite restricted (e.g., *Margaritifera margaritifera* [Linnaeus 1758]; Taeubert et al. 2010), we still lack fundamental information on host fishes of, for example, the threatened European species *Unio crassus* (Philippson 1788) (Hochwald 1997; von Proschwitz and Lundberg 2004; Taeubert et al. 2012). Time-consuming laboratory experiments can provide information on the “theoretical” suitability of fish species (e.g., Keller and Ruessler 1997), but field surveys of infested fish and subsequent species identification of encysted glochidia are the only means to elucidate relative importance of host species in wild mussel populations (e.g., Berrie and Boize 1985; Blažek and Gelnar 2006).

Previous studies, which mostly studied North American unionoids, have confirmed the power of molecular techniques in both detecting cases of morphological misidentification of adult unionoids (Kandl et al. 2001; McCartney et al. 2009) and elucidating new information on glochidia–fish interactions (White et al. 1996; Kneeland and Rhymer 2008). Mitochondrial markers used in this respect include cytochrome c oxidase subunit I (COI) (e.g., Gustafson and Iwamoto 2005), NADH dehydrogenase subunit 1 (NDI) (e.g., Kneeland and Rhymer 2007), and the 16s ribosomal RNA gene (e.g., McCartney et al. 2009). Basing unionoid identification keys on mitochondrial genes can, however, be problematic due to some of the taxa's unusual, doubly uniparental mode of inheritance, resulting in two different mtDNA genomes in male gonadal tissues (Breton et al. 2009). At least with regard to larval and young samples, for which whole individuals—including male gonadal (precursor) tissue—are being processed, inaccuracies in species identification by means of mtDNA-RFLP keys can therefore not be excluded. Basing the molecular identification key on nuclear DNA regions can bypass this problem. Among these, and due to their high abundance in the genome and intermediate rate of sequence evolution, the internal transcribed spacer (ITS) regions of the ribosomal DNA are particularly useful for species identification of samples with low DNA yield (Mindell and Honeycutt 1990). As a consequence, the ITS-1 region has been used in identification of freshwater mussels of several North American regions (e.g., White et al. 1994). ITS-1 is in fact the marker used in the only present molecular key for some European freshwater mussels (Gerke and Tiedemann 2001).

**Figure 1:**
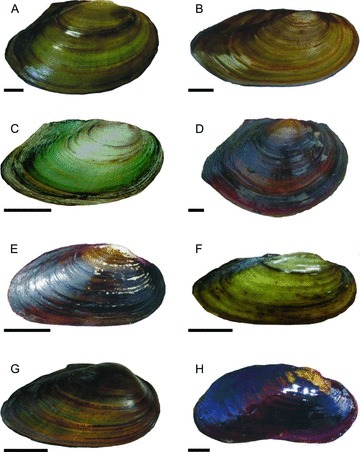
The eight unionoid species of the North and Central European region. (A) *Anodonta anatina*. (B) *Anodonta cygnea*. (C) *Pseudanodonta complanata* (photograph by S. Mueller). (D) *Sinanodonta woodiana*, (E) *Unio crassus*. (F) *Unio pictorum*. (G) *Unio tumidus*. (H) *Margaritifera margaritifera*. Scale bars = 20 mm.

Gerke and Tiedemann's (2001) PCR-RFLP-based key represents a significant aid in the identification of European unionoids. However, several caveats currently impede a quick and reliable identification of unionoid samples from Europe and other regions of the world:

Firstly, Gerke and Tiedemann's (2001) study considers only six of the eight unionoid species of the North and Central European region (Fig. 1). Their identification key does not include the freshwater pearl mussel (*M. margaritifera*), inhabiting cool running waters of the Holarctic region and representing one of the most severely endangered European species (Geist 2010), and *Sinanodonta woodiana* (Lea 1834), a native to Asia, which was introduced to most of Central Europe in the early 1980s, where it is now established and continuing to expand its range (see Pou–Rovira et al. 2009 and references therein). Growing evidence suggests that *S. woodiana* currently may comprise more than one species (Tabe et al. 1994), and it remains unclear which native Asian taxon (taxa) the European form(s) actually belongs to. For example, Soroka (2010) revealed that mitochondrial gene sequences of putative *S. woodiana* from Poland were more similar to South Korean *Anemina arcaeformis* (Heude 1877) than to any Asian *S. woodiana*.

Secondly, the present molecular identification key is based on gene sequences of only northern German individuals. As enzyme restriction sites may be expected to considerably differ in specimens from other European areas, suitability of Gerke and Tiedemann's (2001) key for such non-German samples remains to be tested.

Another issue concerns ubiquitous application of Gerke and Tiedemann's (2001) key on larval samples. As due to their encysted life habit, at least some fish tissue will remain in every glochidial sample, it is crucial to make sure that after DNA extraction, only the glochidial DNA is amplified in PCR. This can be achieved by applying primers that bind exclusively to the mussels’ DNA and not to the host's. While some authors have tested for amplification of host DNA by their respective primers (e.g., Gustafson and Iwamoto 2005), this does not seem to be the case for the ITS-1 primer pairs developed and applied by Gerke and Tiedemann (2001).

Finally, a comprehensive investigation of viable host fish species for given unionoid species or populations necessitates quick processing of several hundreds of infested fish samples. Such large-scale studies are currently hampered by the fact that the respective keys have so far been applied only on single glochidia that were dissected from gills under a light microscope in a rather time-consuming process (Gerke and Tiedemann 2001; Kneeland and Rhymer 2007). Species identification of glochidia from whole fish gill samples would provide a much more practical and rapid approach but has not been established as common practice yet. This is mainly due to the current lack of a systematic assessment of different extraction methods for both fresh and preserved, infested gill samples, although some attempts to extract glochidial DNA from fish gills have been made (Gustafson and Iwamoto 2005). Establishment of a protocol for a quick and reliable extraction of unionoid DNA directly from infested fish gills would considerably speed up species identification of encysted glochidial samples, thereby quickly improving our knowledge of their respective fish hosts.

The main objective of the present study was the development of a molecular identification key for all North and Central European unionoid species, as well as a protocol for glochidial DNA extraction from whole fish gills. This was done by (1) assessing primer pairs developed by Gerke and Tiedemann (2001); and developing and assessing (2) a novel ITS-1 primer pair that allows DNA amplification of all eight unionoid species; (3) a PCR-RFLP identification key; and (4) a protocol for amplification of glochidial DNA directly from whole, infected fish gills.

## Methods

### Samples

Preserved tissue or fresh hemolymph samples of six to 17 adult specimens from all eight species inhabiting North and Central European freshwaters (including two families, i.e., Unionidae and Margaritiferidae) were taken or obtained from various sources and geographic regions (Table 1). Taxa with currently unresolved species status (in particular *Unio* [*pictorum*] *mancus* Lamarck 1819, and *Margaritifera* [*margaritifera*] *durrovensis* Phillips 1928) were not included in the analysis. To ensure accuracy of a priori morphological species identification, we selected only specimens for which species identification was confirmed by all authors and two morphological identification keys (Glöer and Meier–Brook 2003; Killeen et al. 2004). For all samples of the endangered species *M. margaritifera* and *U. crassus*, nonlethal hemolymph sampling as described in Geist and Kuehn (2005) was carried out.

**Table 1:** Number of samples for adult mussels, three host fish species, juvenile mussels, and infested fish gills per site. Ethanol: samples stored in 99% ethanol for three months at 20°C before DNA extraction; fresh: DNA extraction performed immediately after sampling; kit: DNA extraction by Macherey-Nagel NucleoSpin® tissue kit; P-C: DNA extraction by phenol–chloroform extraction method.

Species	Sampling site	Country	Type of water body	Drainage basin	Number of samples
**Adult freshwater mussels**					
Class Bivalvia					
Order Unionoida					
Family Unionidae					
Subfamily Unioninae					
Tribe Anodontini					
*Anodonta anatina* (Linnaeus 1758)	Arbi	Estonia	Lake	Narva	4
	Kieferndorf	Germany	Fish pond	Rhine	2
	Unterpreuschwitz	Germany	Fish pond	Rhine	2
	Aquarium shop Duscher, Passau	Germany	-	-	2
	Ratzteich	Austria	Quarry pond	Danube	2
*Anodonta cygnea* (Linnaeus 1758)	Arbi	Estonia	Lake	Narva	2
	Quidenham Mere	UK	Lake	Great Ouse	2
	Wellsee	Germany	Lake	Schwentine	2
	Wellsee, outflow	Germany	Lake outflow		2
	Unterpreuschwitz	Germany	Fish pond	Rhine	2
	Staffelsee	Germany	Lake	Danube	2
	Kieferndorf	Germany	Fish pond	Rhine	2
	Hornbach gardening and pet shop, Bamberg	Germany	-	-	1
	Ratzteich	Austria	Quarry pond	Danube	2
*Pseudanodonta complanata* (Rossmässler 1835)	Schwentine	Germany	River	Schwentine	4
	Danube	Austria	River	Danube	1
	Morava	Austria	River	Danube	1
*Sinanodonta woodiana* (Lea 1834)	Hornbach gardening and pet shop, Bamberg	Germany	-	-	3
	Hornbach gardening and pet shop, Passau	Germany	-	-	3
	Dehner gardening and pet shop, Nuernberg	Germany	-	-	3
	Pet shop Schmid, Traunstein	Germany	-	-	2
Tribe Unionini					
*Unio crassus* (Philippson 1788)	Schwentine	Germany	River	Schwentine	3
	Ischler Ache	Germany	River	Danube	3
	Donaumoosach	Germany	River	Danube	3
*Unio pictorum* (Linnaeus 1758)	Thames	UK	River	Thames	2
	Schwentine	Germany	River	Schwentine	3
	Ischler Ache	Germany	River	Danube	3
	Staffelsee	Germany	Lake	Danube	1
	Dehner gardening and pet shop, Nuernberg	Germany	-	-	3
*Unio tumidus* (Philippson 1788)	Thames	UK	River	Thames	2
	Schwentine	Germany	River	Schwentine	6
	Aquarium shop Duscher, Passau	Germany	-	-	2
Family Margaritiferidae					
*Margaritifera margaritifera* (Linnaeus 1758)	Hasselån	Sweden	River	Ljungan	2
	Kärmsjöbäcken	Sweden	River	Ångermanälven	2
	Mattarbodbäcken	Sweden	River	Gideälven	2
	Nister	Germany	River	Rhine	2
	Wolfsteiner Ohe	Germany	River	Danube	3
	Aitrach	Germany	River	Danube	2
**Host fish**					
Class Osteichthyes					
Order Salmoniformes					
*Salmo trutta* (Linnaeus 1758)	Hatchery at River Mauka (stock from River Isar)	Germany	-	Danube	4
Order Cyprinodontiformes					
*Leuciscus leuciscus* (Linnaeus 1758)	Glonn	Germany	River	Danube	2
Order Gasterosteiformes					
*Gasterosteus aculeatus* (Linnaeus 1758)	Moosach (flood channel)	Germany	Flood channel	Danube	2
**Juvenile *Margaritifera margaritifera***					
Fresh tissue/kit	Hatchery at TUM (population from River Regnitz)	Germany	-	Elbe	1
Fresh tissue/kit					1 × 3
Fresh tissue/P-C					1
Fresh tissue/P-C					1 × 3
***Salmo trutta* gills infested by *Margaritifera margaritifera* glochidia**					
Fresh tissue/kit	Hatchery at TUM (stock as above)	Germany	-	-	8 ×∼100
Fresh tissue/P-C		Germany			8 ×∼100
Ethanol tissue/kit		Germany			8 ×∼100
Ethanol tissue/P-C		Germany			8 ×∼100

Suitability of different protocols for extracting glochidial DNA from whole, infested fish gill was tested on *M. margaritifera* samples. For this purpose, brown trout (*Salmo trutta* [Linnaeus 1758]) were infected with *M. margaritifera* glochidia following the procedure described in Taeubert et al. (2010). After six months (i.e., few weeks before drop-off), host fishes showed average infection rates of about 100 glochidia on each of the eight gill arches. At this time, four infested fish were sacrificed and all 32 gill arches dissected. To assess effect of tissue preservation on the quality of glochidial PCR product, half of these were processed immediately, while the remaining 16 gill arches were stored in 99% ethanol for approximately three months before proceeding to DNA extraction as outlined below. Suitability of respective DNA extraction protocols for less heavily infested fish gills as those from the wild was tested by evaluating PCR product of two replicates of one and three juvenile *M. margaritifera* (approximately one to three days old), respectively, which were sampled and processed immediately (Table 1).

To test for possible cross-amplification in the nontarget host, three to six tissue samples (fin clips or internal organs) from each of three distantly related host fish species were taken: (1) *S. trutta* (Salmoniformes), the exclusive host of many central European *M. margaritifera* populations (Geist et al. 2006); (2) *Leuciscus leuciscus* (Linnaeus 1758) (Cyprinodontiformes), a host for *Unio* spp. (Berrie and Boize 1985); and (3) *Gasterosteus aculeatus* Linnaeus 1758 (Gasterosteiformes), host for most European unionid species (e.g., Dartnall and Walkey 1979; Berrie and Boize 1985) (Table 1).

### DNA extraction

Mussel hemolymph samples were processed as described in Geist and Kuehn (2005) (i.e., using NucleoSpin Tissue Kit [Machery-Nagel]). The same kit was also used for DNA extraction of uninfested fish samples. For preserved mussel tissue samples, a phenol–chloroform DNA extraction protocol (Sambrook et al. 1999) was followed after washing the samples in distilled water. Since the extraction method applied was previously described to strongly influence the PCR amplification success in mussel shell material (Geist et al. 2008), we tested the suitability of both NucleoSpin Tissue Kit and phenol–chloroform protocol for extracting glochidial DNA (Table 1).

DNA templates for subsequent PCR were prepared by diluting to approximately 50 ng DNA/µl. Since the ratio of glochidial:fish DNA in infested fish gill samples was not known, PCR reactions were performed on undiluted isolated DNA samples.

### Testing different ITS-1 primers

Three ITS-1 primer pairs were tested for exclusive DNA amplification in all eight unionoid species. In a first step, we assessed the two primer pairs developed by Gerke and Tiedemann (2001): (1) their flanking ITS-1 primers (from here on referred to as “ITS-1-GT”); and (2) their internal ITS-1 primers (from here on referred to as “ITS-1-GTi”). For reasons explained in the results section, we then developed (3) a new primer pair for the ITS-1 region with the core objective of perfectly matching sequence regions in all unionoids, but little matches with fish species using the PRIMER3 software (Rozen and Skaletsky 2000), based on published sequences of unionoid and fish species (NCBI database). Primer sequences were ITS-1-F (forward): AGACTGGGTTGCGGAGGT; and ITS-1-R (reverse): CGAGTGATCCACCGCTTAGA.

The three PCRs were performed on all samples, respectively, in 30 µL reactions with the following components: 1× BD PCR reaction buffer (stock solution 10×: 0.8 M Tris-HCl, 0.2 M [NH_4_]_2_SO_4_) (Solis Biodyne), 0.2 mM of each dNTP in mixture (Solis Biodyne), 2.5 mM MgCl_2_ (Solis Biodyne), 0.2 ρmol/µl of each oligonucleotide primer (Biomers), 0.04 U/µl Taq DNA-polymerase (Solis Biodyne), 2.0 µl template DNA (working solution), and HPLC H_2_O (Roth) to adjust to the final concentration. The reaction was performed in a UNO-II cycler (Biometra) with following cycling parameters: one cycle at 94°C for 3 min; 34 cycles of 94°C for 30 sec, 55°C for 30 sec, 72°C for 30 sec; and one cycle at 72°C for 10 min. PCR products were separated on 2.2% agarose gels and visualized under an ultraviolet lamp.

### Developing and testing molecular identification key

The new RFLP identification key was developed by examining restriction sites of partial ITS-1 nucleotide sequences of the eight North and Central European unionoid species using the program NEBcutter V2.0 (http://tools.neb.com/NEBcutter2/index.php). As respective sequences for European representatives of six of the species had already been published (GenBank, Accession Numbers. AJ295287–AJ295292, and DQ060177–DQ060192), we sequenced PCR products for the remaining two species, that is, *M. margaritifera* and *S. woodiana* (published in GenBank under Accession Numbers JN860929 and JN860930). Given the current uncertainties with regard to the species status of European “*S. woodiana*,” we used program BLAST (http://blast.ncbi.nlm.nih.gov/Blast.cgi) to compare our putative *S. woodiana* sequence with published sequences of Asian representatives of this species (comprising three subspecies; i.e., *S. woodiana woodiana*, EU580109, AY484956, GQ393015, GQ393016; *S. woodiana elliptica*, GQ393011–GQ393014; and *S. woodiana pacifica*, GQ393009, GQ393010, GQ393017–GQ393019, AY484957) as well as *A. arcaeformis* (AY484953, AY484954).

Reliability of the new PCR-RFLP key was tested on amplified ITS-1 fragments of all adult mussel samples by performing respective enzyme digestion steps in 12 µl reaction batches: 0.5 U restriction enzyme, 1× buffer R (stock solution 10×: 10 mM Tris-HCl, pH 8.5; 10 mM MgCl_2_; 100 mM KCl; 0.1 mg/mL BSA) (all by MBI Fermentas) and 10.0 µl PCR product. Each batch was incubated for at least 6 h at the optimal temperature of the respective enzyme. Digested fragments were separated on 2.2% agarose gels and visualized under ultraviolet light.

Finally, we tested if the ITS-1 fragment amplified from glochidia was indeed equivalent to that amplified from adult *M. margaritifera*. For this purpose, we compared digested PCR products of DNA samples from glochidia–fish, adult mussel tissue, and theoretically expected band patterns obtained in NEBcutter V.2.0.

## Results

### Application of ITS-primers

The primer pair ITS-1-GT by Gerke and Tiedemann (2001) amplified a segment of 479–565 bp in the seven unionid species under study (Table 1). However, in all tested *M. margaritifera* (family Margaritiferidae) specimens, this primer amplified several DNA segments, one of which was >2000 bp long and thus did not represent the ITS-1 region. In addition, one individual of *Anodonta cygnea* (Linnaeus 1758) from Austria exhibited two bands after amplification with this primer pair, and one to three strong bands were obtained after amplification of all fish samples. Primer ITS-1-GT thus also bound on nontarget host DNA, which also resulted in one to several bands of *M. margaritifera* glochidia/*S. trutta* gill samples.

Amplification with primer pair ITS-1-GTi resulted in one band in all unionid species, whereas multiple bands were obtained for both *M. margaritifera* and all fish species.

**Figure 2:**
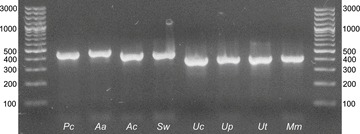
PCR products of adult specimens of eight North and Central European unionoid species applying the ITS-1 primers developed in this study. *Pc*, *Pseudanodonta complanata* (River Schwentine, Germany; 440 bp); *Aa*, *Anodonta anatina* (Fish pond at Kieferndorf, Germany; 460 bp); *Ac*, *Anodonta cygnea* (Quarry pond “Ratzteich,” Austria; 433 bp); *Sw*, *Sinanodonta woodiana* (Germany; 435 bp); *Uc*, *Unio crassus* (River Ischler Ache, Germany; 372 bp); *Up, Unio pictorum* (River Ischler Ache, Germany; 379 bp); *Ut*, *Unio tumidus* (River Thames, UK; 393 bp); *Mm*, *Margaritifera margaritifera* (River Wolfsteiner Ohe, Germany; 404 bp).

**Figure 3:**
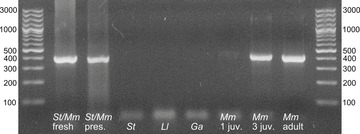
PCR products (using the ITS-1 primers developed in this study) of one *Salmo trutta* gill arch infested with approximately 100 *Margaritifera margaritifera* glochidia (*St*/*Mm* fresh, DNA extraction from fresh material; *St*/*Mm* preserved, DNA extraction from material preserved in 99% ethanol for approximately three months); three host fish species of European freshwater mussels (*St*, *Salmo trutta*; *Ll*, *Leuciscus leuciscus*; *Ga*, *Gasterosteus aculeatus*); one juvenile (*Mm* 1 juv.) and three *Margaritifera margaritifera* juveniles (*Mm* 3 juv.); and an adult sample of *Margaritifera margaritifera* (River Wolfsteiner Ohe, Germany; 404 bp).

The new ITS primers developed in the present study, on the other hand, universally amplified one single and specific segment of the ITS-1 region (372–460 bp) in all samples of the eight unionoid species (Fig. 2). No bands were visible after amplification of fish samples and single *M. margaritifera* juveniles, respectively (Fig. 3). On the other hand, one strong band of the same length as adult *M. margaritifera* samples was obtained after PCR of three juveniles and all infested fish gills. Thus, both DNA extraction methods tested (i.e., Nucleo-Spin Tissue Kit and phenol–chloroform method) successfully extracted DNA from whole fresh and preserved infested fish gill arches with a success rate of 100% (*n*= 32).

### Development and testing of new PCR-RFLP identification key

**Figure 4:**
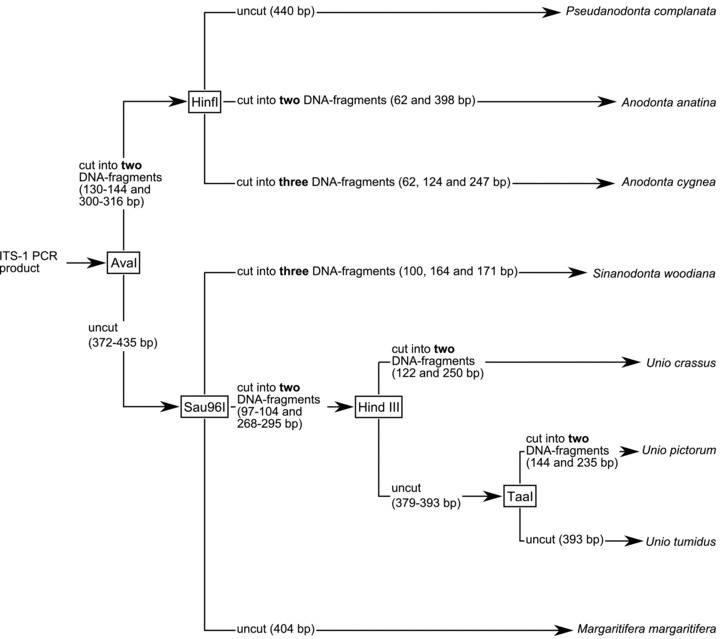
A PCR-RFLP-based molecular identification key to all North and Central European freshwater mussel species. Squares indicate single restriction endonuclease digestions.

**Figure 5:**
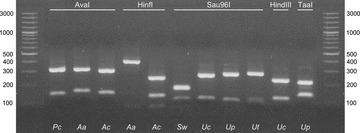
RFLP bands after enzymatic digestion of ITS-1 PCR products for adult specimens of eight North and Central European unionoid species; steps as given in Figure 4. *Pc*, *Pseudanodonta complanata* (River Schwentine, Germany); *Aa*, *Anodonta anatina* (Fish pond at Kieferndorf, Germany); *Ac*, *Anodonta cygnea* (Quarry pond “Ratzteich”, Austria); *Sw*, *Sinanodonta woodiana* (Germany); *Uc*, *Unio crassus* (River Ischler Ache, Germany); *Up, Unio pictorum* (River Ischler Ache, Germany); *Ut*, *Unio tumidus* (River Thames, UK); *Mm*, *Margaritifera margaritifera* (River Wolfsteiner Ohe, Germany). Note that the upper band in *Sw* comprises two products (171 and 164 bp) which are not visually resolved on the gel, and the lowest (smallest fragment size) bands after HinfI digestion in *Aa* and *Ac* are typically faint due to their small size (62 bp).

The new molecular identification key developed allowed determination of all North and Central European species by digestion of the ITS-1 PCR product with five restriction endonucleases (AvaI, HinfI, Sau96I, HindIII, TaaI) in two to four single digestion steps (Fig. 4). Confirming reliability of this PCR-RFLP-based identification key, all morphologically identified adult unionoid samples were assigned to the correct species with 100% accuracy by restriction banding patterns (Fig. 5). No ambiguous restriction patterns were observed. Suitability of glochidial PCR products for RFLP-based species identification was additionally confirmed by the fact that after digestion with HinfI, banding patterns of glochidial products were identical to those of adult *M. margaritifera*.

Finally, comparisons of our *S. woodiana* sequence with the 14 available sequences of Asian representatives of this taxon revealed 99% identities and 0% gaps in all cases. In comparison, only 91% identities and 4% gaps were found when compared to two *A. arcaeformis* sequences from China.

## Discussion

Species determination by means of molecular techniques can bypass the problems of interspecific convergence and intraspecific variability, often impeding accurate morphological identification of freshwater mussels (Watters 1994; Zieritz et al. 2010). However, with regard to North and Central European unionoids, molecular species identification has so far been constrained by shortcomings of the only key available, which does not cover all species of this area. The present study provides the first complete molecular identification key for the North and Central European Unionoida, facilitating quick, low-cost, accurate, and reliable determination of both adult and larval specimens. Despite comprising two more species than the previous study on this topic (Gerke and Tiedemann 2001), our key does not require additional enzyme digestions steps for identification of a given specimen.

In addition, the new ITS-1 primer and key developed offers a more reliable method than Gerke and Tiedemann's (2001). Although our results confirmed suitability of Gerke and Tiedemann's (2001) RFLP key to unambiguously discriminate five European species of the family Unionidae, we observed several bands after PCR of *M. margaritifera* (family Margaritiferidae) and *A. cygnea* samples applying primer pairs ITS-1-GT and ITS-1-GTi. These observations not only indicate the limitation of Gerke and Tiedemann's (2001) key to assess samples that may contain either of these species, but also highlight the importance of including specimens from a wide spatial range when developing a molecular key for a given geographical region.

Besides providing a reliable means for identification of adult samples, the present key can also be applied to larval samples, ultimately providing much needed information on mussel–fish interactions. Our observation of Gerke and Tiedemann's (2001) primer pairs amplifying DNA of three distinctly related host fish species were unexpected as these authors did not report any multiple banding patterns after PCR of glochidia–fish samples. The reason for this discrepancy remains speculative since no information on the host fish species analyzed was provided in that work. However, while previously developed primers were not suitable for identification of encysted glochidia in our experiments, our new ITS-1 primer pair did not amplify host fish DNA and can thus be considered suitable for application on encysted glochidial samples.

Additionally, our study provides protocols for DNA amplification of glochidia from whole infested fish gills. Both DNA extraction methods applied on both fresh and ethanol preserved samples generated PCR products that were of sufficiently high quality to allow for subsequent species identification by means of RFLP banding patterns. Although the artificially infected gill arches used in our experiments carried approximately 100 glochidia, successful amplification of samples of only three recently metamorphosed juvenile mussels indicates that this method can also be applied on less heavily parasitized gills such as those from wild fish populations. As margaritiferid larvae are smaller than those of any other unionoid family (Wächtler et al. 2001), we expect our protocol to work well with regard to any other unionoid species. In a next step, quantitative analyses of infection rates using qPCR approaches can be developed based on this study. However, the applicability of the key under mixed infection scenarios (i.e., mussel larvae from several species on the same host) needs further testing.

Anthropogenic factors such as destruction or deterioration of habitats and host fish populations have resulted in considerable declines in freshwater mussel species (Bogan 1993; Geist 2010). As a result, several European unionoids are now under legal protection and subject to various efforts toward their protection, including habitat restoration and restocking of viable fish hosts (e.g., Altmueller and Dettmer 2006; Zettler and Jueg 2007; Taeubert et al. 2010). As such measures are always based on an initial assessment of the mussel community in question, misidentification of species can result in serious misinterpretation of a given population's vulnerability and ultimately lead to inadequate conservation efforts. Application of this quick and cost-effective PCR-RFLP-based identification key can prevent such cases of misidentifications, thereby ensuring that the limited funds available for mussel conservation are put to their best possible use.

Our key can furthermore be used to detect and survey the nonnative unionoid species *S. woodiana*. This species sometimes resembles European anodontines, has become a serious competitor of native bivalves in several European freshwaters (Kraszewski 2007), and can profoundly alter ecosystem dynamics (Soroka and Zdanowski, 2001). However, recent genetic evidence, such as results by Soroka (2010), suggest that at least some putative European *S. woodiana* populations may in fact belong to a different Asian taxon. Contrary to this author's observations on Polish specimens, the fact that partial ITS-1 sequences of German and Asian *S. woodiana* were almost identical, strongly supports the validity of this taxon name with regard to the specimens analyzed in the present study. While the possibility that putative European *S. woodiana* populations comprise more than one species remains, we expect that the present identification key can in fact assist in quickly detecting such nonnative, non-*S. woodiana* specimens.

Finally, the newly developed methodology of identifying encysted glochidia from whole host fish gills, for the first time, allows comprehensive, large-scale assessments of identities and relative importance of viable host fish species of wild unionoid populations. We expect that by employing this new approach, freshwater ecologists, environmental consultants, and governmental biologists worldwide will be able to quickly assemble a large amount of new data on mussel–fish interactions. The sensitive methodology provided here can thus be universally applied on all unionoid life stages, and has not only the potential to aid in the conservation of these animals but ultimately also in sustaining the crucial ecosystem functions they fulfill.
